# COVID-19 and Decision-Making for Pregnant Women: Taking or Relinquishing Control in Response to a Pandemic

**DOI:** 10.1155/2022/6436200

**Published:** 2022-05-18

**Authors:** Elizabeth Kaselitz, Chelsea Finkbeiner, Sarah Javaid, Sarah Barringer, Sarah D. Compton, Maria Muzik, Cheryl A. Moyer

**Affiliations:** ^1^University of Michigan Medical School, 1301 Catherine Street, Ann Arbor, MI, USA 48109; ^2^Global REACH, University of Michigan Medical School, 1111 E. Catherine Street, Ann Arbor, MI, USA 48109; ^3^School of Public Health, 1415 Washington Heights, University of Michigan, Ann Arbor, MI, USA 48109; ^4^University of Michigan, Ann Arbor, 500 S. State Street, Ann Arbor, MI, USA 48109; ^5^Department of Obstetrics & Gynecology, 1500 E. Medical Center Drive, University of Michigan, Ann Arbor, MI, USA 48109; ^6^Department of Psychiatry, 1500 E. Medical Center Drive, University of Michigan, Ann Arbor, MI, USA 48109; ^7^Department of Learning Health Sciences, 1111 E. Catherine Street, University of Michigan, Ann Arbor, MI, USA 48109

## Abstract

COVID-19 has uniquely impacted pregnant women. From the initial unknowns about its virulence during pregnancy, to frequent and rapidly changing hospital guidelines for prenatal care and delivery, pregnant women have felt intense uncertainty and, based on recent research, increased anxiety. This study sought to determine the impact COVID-19 had on women's birth plans. Open-ended qualitative responses from an anonymous, online survey of pregnant women in the United States, conducted on April 3-24, 2020, were analyzed using the Attride-Stirling qualitative framework. A conceptual framework for understanding the impact of COVID-19 on women's birth plans was generated. 2,320 pregnant women (mean age 32.7 years, mean weeks pregnant 24.6 weeks) responded to the open-ended prompts, reflecting the following themes: the impact(s) of COVID-19 on pregnant women (including unanticipated changes and uncertainty), the effect of COVID-19 on decision-making (including emotional reactions and subsequent questioning of the healthcare system), and how both of those things led women to either exercise or relinquish their agency related to their birth plan. These findings indicate that the changes and uncertainty surrounding COVID-19 are causing significant challenges for pregnant women, and absent more clarity and more provider-driven support, women seeking to cope are considering changes to their birth plans. Health systems and providers should heed this warning and work to provide pregnant women and their families with more information, support, and collaborative planning to ensure a positive, healthy birth experience, even during a pandemic.

## 1. Introduction

COVID-19 has uniquely impacted pregnant women. From the initial unknowns about its virulence during pregnancy, to frequent and rapidly changing hospital guidelines for prenatal care and delivery, pregnant women have felt intense uncertainty and, based on recent research, increased anxiety [1–6].

In the absence of reliable data on the impact of SARS COV-19 on pregnant women in the early days of the pandemic, similar illnesses such as H1N1 influenza were used to model potential outcomes. Yet H1N1 influenza has significantly higher morbidity and mortality among pregnant women than nonpregnant women [7], and it was these data that drove emerging responses.

At the same time, providers and hospitals were facing shortages of personal protective equipment (PPE), fears of being overwhelmed with COVID-19 cases, recommendations to cancel all non-emergent procedures, and indications that limiting visitors could help reduce unnecessary exposures. In this context, pregnant women still needed prenatal, delivery, and postnatal care.

To understand the experience of pregnant women during these early days of COVID-19, we launched an anonymous, online survey. This manuscript describes a qualitative supplement from our larger study [1] exploring the influence of COVID-19 on pregnant women's delivery plans.

## 2. Materials and Methods

### 2.1. Study Design

The methods for the larger study are detailed elsewhere [1], but briefly, this was an anonymous, cross-sectional online survey of English-speaking pregnant women identified and recruited via Twitter, Facebook, and other pregnancy-related online professional and peer communities. Data were gathered from April 3 to 24, 2020. The survey asked pregnancy-related background questions (e.g., weeks pregnant, prenatal care use, and previous births) and basic demographic questions.

### 2.2. Data Sources/Measurement

Data were collected using Qualtrics. Qualitative data were derived from the following open-ended questions:

- “Can you tell us more about how your birth plans have been affected? How have your desires and plans for birth changed, and how have they remained the same?”
- “Is there anything else you would like to tell us about your experiences with pregnancy related to COVID-19?”

### 2.3. Data Analysis

All text data were read by the lead researcher (CF), with substantial portions read by the additional members of the research team (CM, SC, and EK). Using the Attride-Stirling Framework for qualitative analysis [8], a preliminary codebook was developed and revised throughout the coding process. Basic themes were discussed and grouped into organizing themes and global themes, which were then used to generate a conceptual framework. The COREQ criteria were used to guide this research [9].

### 2.4. Ethical Review

This study was deemed exempt from ongoing review by the University of Michigan Institutional Review Board (HUM00179610). This study met IRB exemption status because the subject interaction only involves survey procedures, the identity of human subjects cannot be readily ascertained, and human subjects' responses do not place subjects at risk.

## 3. Results

Table 1 illustrates the demographics of our sample, reflecting women who responded to at least one of the open-ended prompts (*N* = 2320). Overall, women averaged 32.7 years old and 24.6 weeks pregnant, and 78% had a previous birth. More than half were educated beyond college, and 88.9% of the sample was white.

**Figure 1:**
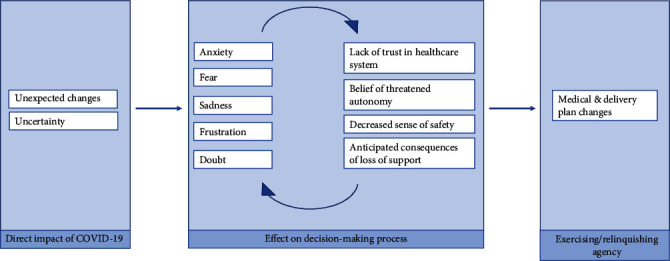
Conceptual framework illustrating the impact of COVID-19 on pregnant women's agency.

Figure 1 illustrates the three dominant organizing themes identified: the direct impact(s) of COVID-19 on pregnant women, the effects of COVID-19 on decision-making, and how both led women to either exercise or relinquish their agency related to their birth plan.

### 3.1. Direct Impact(s) of COVID-19 on Pregnant Women

**Figure 2:**
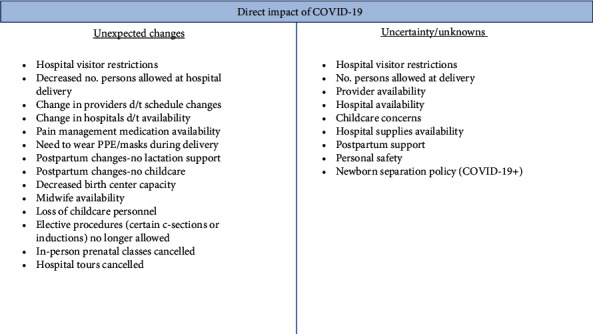
The direct impact(s) of COVID-19 on pregnant women: unexpected changes and uncertainty.

Many respondents described *unexpected changes* and feelings of *uncertainty* caused by COVID-19.  Figure 2 lists the myriad of changes described by respondents, most having to do with the limiting and/or loss of supportive care practices for pregnant or postpartum women—including pain management, lactation support, birth partner support, prenatal classes, and hospital tours.

The examples of changes include the following:

*The biggest change so far is losing our at-home support system because of self-isolation. Not certain who will watch our current children.* (41 years old, 3 previous births)

*It was always my plan to have my husband come with me to the hospital. However, due to daily changes in policies and procedures, our birth plan may need to change.* (30 years old, 1 previous birth)

Women also reported many sources of uncertainty—including what care and support will be available, what hospital restrictions will be in place at the time of delivery, and how hospitals will be able to keep women and newborns safe. While unexpected changes and uncertainty are closely related, specific examples of uncertainty include the following:

*I may have to deliver alone with no support person. With my due date three weeks away, there's still a chance that things will change. I may not be able to go to the birthing center at all. I might not have a doctor. With no ultrasound and no in-person visits where I can be checked, I don't know if the baby is in the right position or anything.* (43 years old, 1 previous birth)

*I have no idea what the situation will look like when I am due to deliver. The unknown is scary.* (33 years old, 1 previous birth)

### 3.2. Effects of COVID-19 on Decision-Making

As illustrated in Figure 1, women reported a host of emotional reactions to the changes and uncertainty sparked by COVID-19–most often expressed as *anxiety*, *fear*, *sadness*, *frustration*, and *doubt*. Such emotional reactions seemed to be both fueled by and contribute to negative perceptions related to trust, support, and safety, including *a lack of trust in the healthcare system*, *beliefs of threatened autonomy*, *a decreased sense of safety*, and *anticipated consequences of loss of support*.

Emotional reactions were hallmarked by words such as “worried,” “terrified,” “afraid,” and “concerned,” such as the following first-time mother articulated:

*My hospital may become a designated COVID hospital. I am considering home birth, but with gestational diabetes I don't know if it's a good idea. It's my first birth. I am terrified.* (33 years old, first pregnancy)

Such emotions often precluded comments regarding women's trust in the healthcare system:

*This is the first time ever that I even thought about a home birth. In normal circumstances, I know the data and would not be willing to accept the risks of a home birth. Now I'm not sure whether the hospital or home birth would have more risks. I don't trust the information that I am getting from healthcare providers, the CDC, or anywhere else.* (34 years old, 1 previous birth)

Other women expressed concerns about the quality of care that might be delivered in the midst of a pandemic:

*I don't feel care will be up [to] the usual standard and am worried about medical errors due to exhausted staff.* (26 years old, first pregnancy)

*I am concerned that my scheduled induction will be canceled due to lack of beds and resources.* (38 years old, 2 previous births)

Many women described frustration with their lack of control over nearly every aspect of their impending delivery, which some experienced as a threat to their autonomy:

*I have no plan anymore because planning for something that feels so out of my control makes me extremely anxious.* (30 years old, first pregnancy)

*Even though I am afraid to give birth in a hospital, I am even more afraid to give birth in a different setting. I am too far along to rethink my plan, in my opinion.* (34 years old, first pregnancy)

Many women described COVID-19 decreasing their sense of safety, which overlaps with the emotional themes of anxiety, fear, and doubt:

*I am terrified that we will get exposed in the hospital, and our newborn baby will be at risk. If delivering at the hospital, I am planning to ask everyone that enters the room to wear protection (especially mask) and wash hands. I am considering a natural birth in hopes that we can deliver and be discharged ASAP to minimize exposure.* (35 years old, 1 previous birth)

In addition, women anticipated a loss of support around the time of delivery as a result of COVID-19. While this theme overlaps with COVID-19-related changes and uncertainties, as well as some of the emotional impact of such uncertainties, many women wrote about anticipating a loss of support, and what that might mean for their birth plans:

*If the rules get more strict to the point where they say my husband is unable to be with me during birth, I feel like I have no other option but to have a home birth.* (27 years old, first pregnancy)

### 3.3. Exercising or Relinquishing Agency

**Figure 3:**
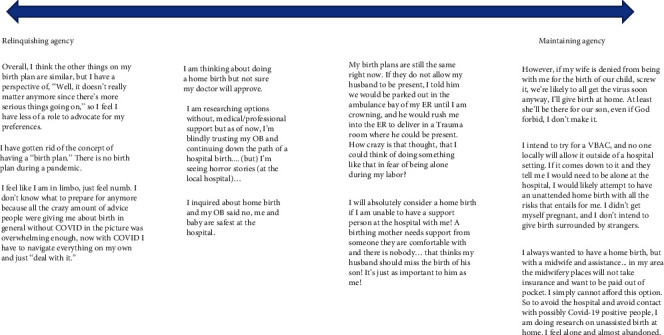
The spectrum of women's responses to COVID-19: relinquishing to maintain agency.

Figure 1 illustrates how the direct and indirect impacts of COVID-19 ultimately lead women to consider their own role in whether or not they could impact their delivery experiences. While uncertainty and heightened emotions drove some women to seek aspects of the birth experience that they could control (exercising agency), the same uncertainty and heightened emotions drove other women to surrender their sense of control over the birth process (relinquishing agency). The spectrum of responses related to exercising or relinquishing agency is outlined in Figure 3.

The left side of Figure 3 represents women who described relinquishing their agency with regard to delivery during COVID-19:

*I have gotten rid of the concept of having a ‘birth plan'. There is no birth plan during a pandemic.* (32 years old, 1 previous birth)

Comparatively, the respondents on the right side of Figure 3 represent those who have opted to exercise their agency, often describing moving away from a hospital birth for more control, such as the following:

*I had never considered home birth before, but if the situation continues to escalate, I will look into it for fear of being forced to deliver without a support person or generally losing control of the most important things in my birth plan.* (30 years old, first pregnancy)

There are a number of responses in between these two ends of the spectrum, with women describing contingency plans in the event their partners are not allowed with them during delivery. As one respondent stated:

*If they do not allow my husband to be present, I told him we would be parked out in the ambulance bay of my ER until I am crowning and he would rush me in the ER to deliver in a trauma room where he could be present.* (27 years old, first pregnancy)

Concerns about not having a birthing partner were pervasive and seemed to be the tipping point for some women on whether they would relinquish control or exercise agency while giving birth during a pandemic.

## 4. Conclusions

This study highlights the uncertainty and unexpected changes pregnant women experienced during the COVID-19 pandemic, as well as the variability of how women responded. Responses ran a gamut of emotional reactions, ultimately leading women to consider the potential impact of COVID-19 on their delivery plans, and then either surrender their agency or attempt to exert agency by creating a birth plan that could be maintained in the face of uncertainty.

These findings illustrate the intense disruption that pregnant women were experiencing in the early months of the pandemic, as well as their pervasive anxiety about giving birth during this time. Some coped by disengaging (“there is no such thing as a birth plan during a pandemic”), while others coped by seeking to control whatever they could about their birth experience (“we (will park) outside the ER…until I am crowning”).

Seeking control over the birth experience is nothing new—it has arguably just become more acute during the COVID-19 pandemic. Aarthun and Akerjordet [10] discuss the evolution of healthcare and the shift away from a paternalistic model to more shared decision-making between physicians and patients. Since COVID-19 emerged, women went from having the ability to put together detailed birth plans and the option of multiple sources of delivery-room support to strict (and changing) rules about what their experience can or cannot include. Women also faced potential quarantine from their newborns after delivery, possibly the ultimate loss of control.

Similar to our findings, Gildner and Thayer [11] found pregnant women to be making changes in response to COVID-19—with 45.2% of their respondents reporting some change in their birth plan because of their concerns. They also similarly reported that some respondents moved away from having hospital births as a result of the pandemic.

Our findings indicate that some women have consciously decided to retain agency over their healthcare decision-making during this crisis. However, this does not necessarily mean that those who claim more agency are necessarily less anxious. In our larger study [1], we found that women who reported planning to change their birth plan away from a hospital birth had greater increases in their levels of anxiety than those who did not make that decision. Thus, it is possible that those who are most anxious are most likely to report seeking to retain agency and further research is needed to determine if actually retaining agency ultimately reduces anxiety.

Levy [12] explored informed decision-making for pregnant women and found that “maintaining equilibrium”—or preserving balance by managing worry and distress—most often drove pregnancy-related choices. Levy found the reaction of mothers ranged from fully “asserting” their choices to “handing over” decision-making to midwives, and these reactions were often influenced by levels of trust in information and providers [12]. Those who asserted their choices often felt respected by and had trust in their providers, while those who fully handed over decision-making sometimes grappled with mistrust and fear of authority. More research is warranted to further understand the relationship between agency and decision-making for pregnant women during the COVID-19 pandemic, as well as if certain provider dynamics potentially drove women away from facility deliveries. Regardless, what is certain is that hospitals and providers can do better to make women feel safe and cared for during this crisis.

This research reflects the thoughts of more than 2,000 women throughout the U.S. during the early ramp-up of the COVID-19 pandemic, reflecting a geographically and educationally diverse pool of respondents expressing surprisingly similar sentiments. The strengths of this research include the sample size, the ability to hear women describe concerns in their own words, and the robust analysis that involved manually assessing thousands of responses. Nonetheless, these cross-sectional, self-reported data, while collected at a pivotal time in the escalation of COVID-19 in the U.S., preclude our ability to determine whether women did indeed relinquish control over their birthing plan or whether they were able to exert agency over their deliveries. Future research that explores women's actual birth experiences during the COVID-19 pandemic is warranted.

These findings indicate that COVID-19 caused significant challenges for pregnant women, and absent more clarity and more provider-driven support, women seeking to cope considered changing their birth plans. Health systems and providers should work to provide pregnant women and their families with more information, support, and collaborative planning to ensure a positive, healthy birth experience, even during a pandemic.

## Figures and Tables

**Table 1:** Sample demographics (*N* = 2320).

Variable	Mean (SD)
Maternal age	32.75 (4.5)
Weeks pregnant	24.65 (9.5)
Number of previous births	*N* (%)
0	403 (22.0%)
1-2	1309 (71.4%)
3+	121 (6.6%)
Highest level of education	
High school or less	143 (6.2%)
College or less	985 (42.5%)
Master's degree	613 (26.5%)
Postgraduate degree	576 (24.8%)
Residence	
Urban	593 (25.6%)
Peri-urban	1346 (58.1%)
Rural	377 (16.3%)
Marriage status	
Single	60 (2.6%)
Married	2057 (88.9%)
Divorced/separated/widowed	15 (0.7%)
Committed relationship	182 (7.9%)
Ethnicity	
Hispanic/Latino	147 (6.4%)
Race	
White	2036 (88.9%)
Black/African-American	53 (2.3%)
Other	141 (6.1%)
Mixed	73 (3.2%)
Original birth plan	N (%)
To give birth in a hospital	2237 (96.4%)
To give birth in a birth center outside a hospital	27 (1.2%)
To give birth at home	38 (1.6%)
I do not know where I am going to give birth	18 (0.8%)
Post-COVID-19 birth plan	
To give birth in a hospital	2032 (87.6%)
To give birth in a birth center outside a hospital	33 (1.4%)
To give birth at home	69 (3.0%)
I do not know where I am going to give birth	185 (8.0%)

## Data Availability

De-identified data sets are available upon request.
